# Nurses' and Nursing Students' Knowledge and Attitudes regarding Pediatric Pain

**DOI:** 10.1155/2015/210860

**Published:** 2015-10-12

**Authors:** Mario I. Ortiz, Héctor A. Ponce-Monter, Eduardo Rangel-Flores, Blanca Castro-Gamez, Luis C. Romero-Quezada, Jessica P. O'Brien, Georgina Romo-Hernández, Marco A. Escamilla-Acosta

**Affiliations:** ^1^Área Académica de Medicina del Instituto de Ciencias de la Salud de la Universidad Autónoma del Estado de Hidalgo, 42090 Pachuca, HGO, Mexico; ^2^Universidad del Futbol y Ciencias del Deporte, 42162 San Agustín Tlaxiaca, HGO, Mexico; ^3^Hospital del Niño DIF Hidalgo, 42083 Pachuca, HGO, Mexico; ^4^Escuela Americana de Pachuca, 42092 Pachuca, HGO, Mexico

## Abstract

Nursing staff spend more time with patients with pain than any other health staff member. For this reason, the nurse must possess the basic knowledge to identify the presence of pain in patients, to measure its intensity and make the steps necessary for treatment. Therefore, a prospective, descriptive, analytical, and cross-sectional study was conducted to investigate the knowledge and attitudes regarding pediatric pain in two different populations. The questionnaire, Pediatric Nurses Knowledge and Attitudes Survey Regarding Pain (PKNAS), was applied to 111 hospital pediatric nurses and 300 university nursing students. The final scores for pediatric nurses and nursing students were 40.1 ± 7.9 and 40.3 ± 7.5, respectively. None of the sociodemographic variables predicted the scores obtained by the participants (*P* > 0.05). There was a high correlation between the PKNAS scores of pediatric nurses and nursing students (*r* = 0.86, *P* < 0.001). It was observed that the degree of knowledge about pain and its treatment was very low in both groups. Due to this deficiency, pain in children remains inadequately managed, which leads to suffering in this population. It is necessary to increase the continued training in this subject in both areas.

## 1. Introduction

Pain is an individual, multifactorial experience influenced by culture, previous pain events, beliefs, moods, and ability to cope [1]. It may be an indicator of tissue damage but may also be present in the absence of an identifiable cause. The degree of disability in relation to the experience of pain varies; similarly, there is individual variation in response to methods of pain relief [2]. It is of particular importance to nursing care that unrelieved pain reduces patient mobility, resulting in complications such as deep vein thrombosis, pulmonary embolus, and pneumonia [3]. Postsurgical complications related to inadequate pain management negatively affect the patient's welfare and the hospital performance because of extended lengths of stay and readmissions, both of which increase the cost of care [3].

Today, pain in children is not adequately addressed, and yet there is a deficiency of knowledge in the treatment of pain in people of different areas of health, such as physicians, nurses, psychologists, and dentists [4–6]. Medical staff often exhibit widespread and inappropriate attitudes towards pain management in children despite the efficacy of a variety of psychological and pharmacological interventions for reductions the pain [7, 8]. A statistically significant proportion (49–64%) of hospitalized children receives inadequate pain management despite the increase in knowledge and available treatments [9, 10]. For this reason, developed and developing countries have tried to improve and evaluate the preparation of health professionals to provide the best standards of care to children in pain [11]. In this sense, the Pediatric Nurses' Knowledge and Attitudes Survey Regarding Pain (PNKAS) tool was applied to two hundred and seventy-four nurses at a large children's medical center. Sixty-six percent of the questions were answered correctly. Insufficient knowledge was mainly found in the pharmacology of opioids and drugs analgesics [12]. Vincent [13] evaluated nurses' knowledge and attitudes about relieving children's pain and perceived barriers to optimal pain management and analgesics administered by nurses in relation to levels of children's pain. Inadequate or insufficient physician medication orders for pain were identified by 99% of nurses as the greatest barrier to optimal pain management. The mean score was 76% with nurses having knowledge deficits about nondrug methods of pain relief, analgesics drugs, and the incidence of respiratory depression. A modified version of the PNKAS was used by Rieman and Gordon [14] to evaluate nursing competency to manage pain at eight pediatric hospitals. Two-hundred-ninety-five nurses participated in the study. Nurses' individual scores on the PNKAS ranged from 37 to 100% correct, with a mean of 74%. Nursing education, professional activity, and years of clinical experience contributed to the knowledge necessary for competency in pain management, as evidenced by higher scores using this survey tool. The 10 survey questions most often answered incorrectly were related to opioids and analgesics drugs pharmacology. Subsequently, Yildirim et al. [15] examined information about the knowledge and attitudes of Turkish oncology nurses regarding cancer pain management. The average correct response rate was 35.41%, with rates ranging from 5.13% to 56.41% for each survey question. Among the 39 pain knowledge questions assessed, the mean number of correctly answered questions was 13.81 ± 5.02, with a range of 2 to 22 items correctly answered. Huth et al. [16] evaluated the effectiveness of a pain education intervention on Mexican nurses' knowledge and attitudes toward pediatric pain. On a 30-item scale, the most important result was the basal score (preintervention) of 13.1 ± 3.89. This result showed a knowledge deficiency of pain and its management in a group of Mexican nurses. A study on Turkey nurses evaluated the level of knowledge and attitudes of pediatric nurses regarding the child's pain using the PNKAS. The total mean score on the PNKAS scale was 38.2%. The highest score was 65% and the lowest score was 15% [17]. It is likely that the lack of knowledge demonstrated by pediatric nurses from the studies mentioned results from deficiencies in their educational nurse training and preparation and a lack of opportunities to engage in continuing professional development. For this reason, we proposed to conduct a study in two phases to compare the degree of knowledge and attitudes regarding pediatric pain in nurses of a Mexican pediatric hospital and in nursing students of a Mexican university.

## 2. Materials and Methods

### 2.1. Design

A prospective, descriptive, analytical, and cross-sectional study was conducted to investigate the knowledge and attitudes regarding pediatric pain in two different populations: (a) pediatric nurses of a pediatric regional hospital (Mexico) and (b) nursing students of a Mexican university; this last institution is close to the pediatric hospital (these university students go to the pediatric hospital to perform their clinical practices).

### 2.2. Procedure

Conduction of the study was done in two phases. In the first phase, a convenience sample of 111 pediatric nurses was evaluated in the pediatric hospital. These nurses were full-time employees at the hospital. In the second phase, a convenience sample of 300 nursing students was evaluated at the university. Students were attending the last six levels (semesters) of the undergraduate nursing. In the first phase, the researchers got to the hospital and asked the paediatric nurses of the different work shifts (morning, afternoon, night, and special shift) to fully complete the questionnaire (in one application). For the convenience sample of nurses, this was completed during several meetings at the hospital. Participants had 20 minutes to voluntarily complete the questionnaire. The same process was repeated with nursing students during their classes or academic activities in their school.

Permission was obtained to use, modify, and translate the PNKAS into Spanish (permission of the use of PNKAS was obtained directly from Betty R. Ferrell. City of Hope Pain Resource Center, 1500 East Duarte Road, Duarte, CA 91010, 626 256-HOPE Ext. 63829, Email: prc@coh.org) [12]. The questionnaire is a useful and valid tool to assess knowledge and attitudes regarding pediatric pain in nurse's staff of hospitals and in nursing students in different languages such as Italian, English, Mexican, Taiwanese, and Turkish [5, 12, 15–18]. The questionnaire applied consisted of four subsections. The first subsection consisted of 23 questions in which the answer choice was true or false. The second subsection consisted of 13 multiple-choice questions. The third subsection was the presentation of two clinical cases and four reagents. The PNKAS is constituted by these three subsections. Finally, the fourth subsection of the questionnaire corresponded to the ability to identify a series of drugs, which of them are opioids and which are not.

### 2.3. Ethical Considerations

The study protocol was approved by the Ethics and Investigation Committees of the Hospital del Niño DIF (Pachuca, Hidalgo, Mexico) and the study was carried out according to the guidelines delineated by the Declaration of Helsinki. Informed consents were obtained for completion of the questionnaires from all participants. Anonymity was assured and emphasized.

### 2.4. Data Analysis

Data was entered into a computerized database. SPSS version 17 for Windows (SPSS Inc., Chicago, IL, USA) was used for descriptive and inferential statistical analyses. We performed exploratory analysis using the Pearson Chi-square test. Knowledge and attitudes about pain were analyzed with logistic regression analysis. Test scores were considered to be dependent variables, while nurses' sociodemographics were potential predictors. For the multivariable analysis, we used stepwise logistic regression analysis. The *t*-test was used to examine any difference between comparison groups. The relationship between the pediatric nurses and nursing student scores was analyzed by the Pearson coefficient. The significance level was set at *P* < 0.05.

## 3. Results

### 3.1. Sociodemographic Data of Pediatric Nurses

In the evaluation, 111 pediatric nurses volunteered to participate in the study. The mean age ± SD of these participants was 30.2 ± 7.4 years. One hundred and five participants were women (94.6%) and six (5.4%) were men.  Table 1 shows the baseline characteristics of the pediatric nurses. No variable related to baseline characteristics of the pediatric nurses showed significant correlation or association with the PNKAS or opioid knowledge scores (*P* > 0.05).

### 3.2. PNKAS Scores of Pediatric Nurses

The five questions most often answered correctly by participants in the survey are presented in Table 2. The five questions most often answered erroneously are displayed in Table 3. On a 40-item scale, an average score of correct answers of 16.0 ± 3.2 was obtained, with a minimum of nine correct answers and a maximum of 25 correct answers. To observe and understand these scores, answers were analyzed as follows: standard scores were derived by applying the following equation: average of correct score/total score × 100. Performing this conversion, a standard average score of 40.1 ± 7.9 was obtained, with a minimum score of 22.5 and the highest rating of 62.5.

### 3.3. Knowledge about Opioids of Pediatric Nurses

The knowledge about which of a series of drugs are or are not opioids or opiates is shown in Table 4. On a 12-item scale, an average score of correct responses of 8.0 ± 3.8 was obtained, with a minimum of zero correct answers and with a maximum of 12 correct answers. From a scale of zero to 10, an average score of 6.7 ± 3.2 was obtained, with a minimum score of 0 and maximum score of 10. None of the independent variables predicted the PKNAS or opioids knowledge scores obtained by the pediatric nurses (*P* > 0.05, Pearson Chi-squared test).

### 3.4. PNKAS Scores of Nursing Students

In the second phase, the questionnaire was applied to 300 nursing students in their third to eighth semester. The mean age of this group of participants was 21.0 ± 1.7 years. Two hundred and sixty-eight participants were women (89.3%) and 32 (10.7%) participants were men.  Table 5 describes the gender and average age of the nursing students. The five questions most often answered appropriately by nursing students are presented in Table 6 and the five questions most often answered incorrectly are displayed in Table 7. On a 40-item scale, an average score of correct answers of 16.1 ± 3.0 was obtained, with a minimum of seven correct answers and a maximum of 26 correct answers. As the previous conversion, an average score of 40.3 ± 7.5 was observed, with a minimum score of 17.5 and the highest rating of 65.0.

There was a high correlation between the PKNAS scores of pediatric nurses and nursing students (Pearson coefficient = 0.86, *P* < 0.001). However, there was no significant statistical difference between the PKNAS scores obtained by the two groups of participants (*P* > 0.05).

### 3.5. Knowledge about Opioids of Nursing Students

On a 12-item scale, an average score of correct responses of 5.96 ± 3.64 was obtained, with a minimum score of zero and a maximum score of 12 (Table 8). On a scale from “0 to 10,” an average score of 4.97 ± 3.03 was demonstrated, with a minimum score of zero and maximum score of 10.

There was moderate correlation between the scores of knowledge about opioids of pediatric nurses and nursing students (Pearson coefficient *r* = 0.67, *P* = 0.018). Likewise, there was a significant statistical difference between the opioids knowledge scores obtained by the pediatric nurses (8.0 ± 3.8) and those obtained by nursing students (5.96 ± 3.64) (*P* = 0001).

Nursing students were also analyzed separately (by semesters). There was no significant statistical difference between the PKNAS or opioids knowledge scores obtained by the six groups of students (data not shown) (*P* > 0.05).

## 4. Discussion

In this study, hospital nurses obtained an average score of correct answers of 16.0 ± 3.2 (on a 40-item scale) or 40.1 ± 7.9% (40 correct answers = 100%) on the PKNAS. This last mean score found in our study was slightly smaller than the preintervention score of 43.7 observed by Huth et al. [16] in Mexican nurses and was smaller than the postintervention score of 55.7 of the same study. In general, the standard score of the pediatric nurses in our study is lower than that observed in nurses of other countries [12–15, 18, 19]. As the problem of pain assessment and management in children persists, despite decades of research, there is an urgent need to develop continual educational initiatives that promote the use of theoretical knowledge in hospital practice. In this sense, an increase of 12% in the pediatric nurses' pain knowledge and attitude score was obtained after a 4-hour educational program in Mexican hospital nurses [16]. Therefore, it is suggested to establish permanent educational and evaluation programs longer than four hours concerning pain assessment and treatment at the hospitals.

Regarding the level of pain (intensity) in the two clinical cases of the questionnaire applied, the correct value of both questions was eight. It was noted that only four of 111 pediatric nurses (3.6%) correctly answered the evaluation of the patient who had a pain level of eight but that the patient was quiet and not complaining (Table 3), while 21 of 111 (18.9%) answered correctly assessing that the patient had a pain level of eight but that the patient was restless and expressed a lot of pain (data not shown). Our results are similar to a study by McCaffery and Ferrell [20], where 456 nurses were asked how to rate the pain in two hypothetical patients, one who had behavioral manifestations and another who had no behavioral manifestations. The patient with the behavioral response had higher scores; this shows that nurses are influenced by patient behavior (as observed in our study) and do not necessarily have behavioral or physical responses to patients with pain.

The choice of drugs and routes of administration are determined according to the type of patient (child, adult, women, etc.), the clinical condition (asthma, hypertension, trauma, postoperative, etc.), and the speed at which you want to initiate and maintain the pharmacological effect (angina pectoris, hypertensive emergency, severe pain, etc.). There is evidence that has shown that nurses have poor knowledge in pharmacotherapy [21–23]. That problem was observed in our study, where the question, “the recommended route of administration of opioid analgesics to children with continuous or persistent pain is?”, was correctly answered by only two pediatric nurses. By contrast, 47 (42.3%) pediatric nurses correctly answered the question, “the recommended route of administration of opioid analgesics to children with brief severe pain of sudden onset, for example, trauma or postoperative pains?” (data not shown), where the route of choice for the treatment of acute and severe pain is the intravenous route. In this same sense, we identified deficiencies in the differentiation of opioid drugs by pediatric nurses. In this case, morphine was recognized as an opioid by 94 (84.7%) nurses; 73 (64.9%) participants recognized nalbuphine as an opioid and 72 (64.9%) nurses recognized fentanyl as an opioid as well. However, many participants had the wrong idea or knowledge that several nonsteroidal anti-inflammatory drugs (NSAIDs) such as indomethacin, ketorolac, and metamizol are opioids. For this reason, it is necessary to give courses in clinical pharmacology to pediatric nurses at the hospital and improved pharmacology courses in bachelor's degrees in nursing.

In regard to the university curricula for health academic areas, such as medicine, nursing, or dentistry, these do not include a subject or “special unit” that focuses on the teaching of the physiopathology, assessment, diagnosis, and treatment of pain. The “pain” issue is only taught lightly in subjects such as physiology, nosology, pathology, surgery, and pharmacology. Nursing students' low scores in studies that evaluated knowledge of pain management may be due to the scarce time that has been devoted to this topic in the nursing curricula. Graffam [24] demonstrated that schools of nursing devoted approximately four hours to pain management in their curricula. Likewise, Ferrell et al. [25] found that nursing schools dedicated about 18.9 hours of clinical time to knowledge and beliefs about pain. On the other hand, Watt-Watson et al. [26] determined the designated time for mandatory pain content in curricula of major Canadian universities for students in health science and veterinary programs before being licensed. Authors found that the average total hours for designated mandatory formal content by nine nursing centers of faculties were 31 ± 4.2 hours (range: 0–48). A recent study explored the depth and breadth of pain content in 3-year preregistration pediatric nursing courses in 56 UK higher education institutions [27]. Authors found that these courses in UK do not always train students to manage pain effectively in clinical practice and they found limited content on pain in nursing curricula. The nursing program of the university evaluated in our study is constituted of eight semesters with 50 courses, grouped into the following four areas: scientific technical, methodological, and social humanistic and basic support. Courses of these areas are distributed in 2,085 hours of theory and 2,460 hours of clinical practices. None of these 50 courses has as its main objective the physiopathology, assessment, and treatment of pain. For this reason, low or very low scores were obtained by the nursing students in the PKNAS (40.3%). This mean score found in our nursing students was almost similar to the mean score obtained of Australian and Philippine nursing students (38.6%) [28]. However, the scores of our nursing students were lower than those reported by Plaisance and Logan (64%) [29], Chiang et al. (55%) [18], and Keefe and Wharrad (54%) [30]. It is important for the nursing schools to consider the possibility of integrating pain education into the conventional nursing curricula in a systematic manner to better prepare students in their future career. Likewise, it is hoped that some deficiencies in nursing education relating to pain would be addressed through experiential learning “on the job” and from mentoring from more senior staff. However, the high correlation of the low scores of the two evaluated groups may indicate that pediatric nurses possess the same deficient knowledge and attitudes regarding pain that were acquired when they were nursing students. Therefore, the poor preparation of the hospital pediatric nurses could worsen or produce no change in the attitude and knowledge of the nursing students about pain management in children. Therefore, it is necessary to develop continuous, comprehensive, and inclusive educational initiatives to improve these aspects in clinical practice.

On the other hand, the nursing program of the university evaluated has only one course called “pharmacology.” It is almost impossible that, in 60 hours (two hours/week of theory and two hours/week of practice) of the course, nursing students can get or learn all the pharmacology of all systems, including the pharmacology of pain, such as NSAIDs, opioids, and anesthetics. This situation was observed in our evaluation, where four of the top five questions answered incorrectly by nursing students are associated with pain pharmacology (Table 7). Also it is observed in our evaluation that nursing students had difficulties differentiating between opioids and nonopioids (Table 8). Our results agree with the results obtained in a study that evaluated the views and knowledge base of graduating nursing students in the area of taking care of children in pain in Finland [31]. The lack of knowledge in nursing student especially in the area of pain medications as well as in the assessment of pain was one of the most important results. Authors mentioned that there is an urgent need for more detailed learning about taking care of children with pain.

One limitation of our study includes the lack of evaluation or quantification of the total hours of the university nursing curricula devoted to the assessment and management of pain. This data could help us to understand and/or justify the low scores found in the nursing students.

As the opportunities that students have to assess and manage children's pain in their clinical practice along the years might vary, another limitation of our study was the recruitment and results analysis of students from six different levels.

## 5. Conclusions and Recommendations

We found that the level of knowledge about pain and its proper management is very poor, in both active pediatric nurses and nursing students. In this regard, the final score of the PKNAS in the pediatric nurses was not superior to or different from that of nursing students, so it follows that both groups have deficiencies. Due to these insufficiencies, pain in children remains inadequately and poorly managed, which leads to unnecessary suffering in the pediatric population. Therefore, it is necessary to increase the capacitation in this subject in both groups of participants. This capacitation must be continuous and include all aspects of the evaluation and treatment of pain in children.

## Figures and Tables

**Table 1:** Baseline characteristics of hospital pediatric nurses.

Time spent by the nurses with patients in pain (%)	
0 to 50	37 (33.3%)
51 to 75	20 (18.0%)
76 to 100	54 (48.6%)
Years of nursing experience	
1 to 5	54 (48.6%)
6 to 10	33 (29.7%)
>10	24 (21.6%)
Years of pediatric nursing experience	
1 to 5	61 (55.0%)
6 to 10	33 (29.7%)
>10	17 (15.3%)
Are you a member of an organization or association of nursing?	
No	108 (97.3%)
Yes	3 (2.7%)
Are you a member of a committee of hospital nursing?	
No	111 (100%)
Does your pediatric facility have a Pain Management Protocol?	
No	66 (59.5%)
Unknown	45 (40.5%)
Does your pediatric facility have a Pain Management Critical Pathway?	
No	57 (51.4%)
Unknown	54 (48.6%)
Does your pediatric facility have a Pain Management Committee?	
No	26 (23.4%)
Unknown	84 (75.7%)
How many professional journals do you read monthly?	
0	71 (64.0%)
1	24 (21.6%)
2 to 4	16 (14.4%)

**Table 2:** Top 5 questions answered correctly by hospital nurses.

Item content (correct answer)	% correct
Analgesia for continuous pain should be given: (around the clock on a fixed schedule)	92.8

After the initial recommended dose of opioid analgesic, subsequent doses should be adjusted in accordance with the individual patient's response. (True)	86.5

Analgesics for post-operative pain should initially be given: (around the clock on a fixed schedule)	86.5

Children who will require repeated painful procedures (i.e. daily wound care or blood draws), should receive maximum treatment for the pain and anxiety of the first procedure to minimize the development of anticipatory anxiety before subsequent procedures. (True)	82.9

Children with pain should be encouraged to endure as much pain as possible before resorting to a pain relief measure. (False)	81.1

**Table 3:** Top 5 questions answered incorrectly by hospital nurses.

Item content (correct answer)	% incorrect
The recommended route of administration of opioid analgesics to children with continuous or persistent pain is: (oral)	98.2

Patient A: Andres is 14 years old and this is his first day after abdominal surgery. As you enter his room, he smiles at you and continues talking and joking with his visitor. Your assessment reveals the following information: BP = 120/80; HR = 80; *R* = 18; on a scale of 0 to 10 (0 = no pain/discomfort, 10 = worst pain/discomfort), he rates his pain as 8. On the patient's record you must mark his pain on the scale below. Circle the number that represents your assessment of Andrew's pain: (8).	96.4

Narcotic/opioid addiction is defined as psychological dependence accompanied by overwhelming concern with obtaining and using narcotics for psychic effect, not for medical reasons. It may occur with or without the physiological changes of tolerance to analgesia and physical dependence (withdrawal). Using this definition, how likely is it that opioid addiction will occur as a result if treating pain with opioid analgesics? (<1%)	95.5

Your assessment, above, is made 2 hours after he received morphine 2 mg IV. Half-hourly pain ratings after the injection ranged from 6 to 8, and he had no clinically significant respiratory depression, sedation, or other side effects. He has identified 2 as an acceptable level of pain relief. His physician's order for analgesia is “morphine IV 1–3 mg q 1 h PRN pain relief”. Check the action you will take at this time: (Administer morphine 3 mg IV now).	94.6

Observable changes in vital signs must be relied upon to verify a patient's statement that he has severe pain. (False)	93.7

**Table 4:** Knowledge of hospital nurses about which of the following drugs is or is not opioid or opiate.

	Correct responses	
	*n*	%
Morphine	94	84.7
Fentanyl	72	64.9
Ibuprofen	82	73.9
Ketorolac	67	60.4
Metamizol	70	63.1
Nalbuphine	73	65.8
Naproxen	81	73.0
Nimesulide	81	73.0
Paracetamol	86	77.5
Indomethacin	45	40.5
Tramadol	60	54.1
Aspirin	83	74.8

**Table 5:** Age and gender of the nursing students of different semesters.

	Semester						Total
	3rd	4th	5th	6th	7th	8th	
*n*	64	56	62	42	40	36	300

*Age*							
Mean ± SEM	19.4 ± 1.0	20.7 ± 1.6	21.2 ± 1.8	21.7 ± 1.4	21.3 ± 1.1	22.9 ± 1.1	21.0 ± 1.7

*Male *							
*n* (%)	4 (6.25)	7 (12.5)	6 (9.7)	6 (14.3)	6 (15.0)	3 (7.5)	32 (10.7)

*Female*							
*n* (%)	60 (93.75)	49 (87.5)	56 (90.3)	36 (85.7)	34 (85.0)	33 (82.5)	268 (89.3)

**Table 6:** Top 5 questions answered correctly by nursing students.

Question (correct answer)	% correct
After the initial recommended dose of opioid analgesic, subsequent doses should be adjusted in accordance with the individual patient's response. (True)	92.0

Children with pain should be encouraged to endure as much pain as possible before resorting to a pain relief measure. (False)	84.3

Analgesia for continuous pain should be given: (around the clock on a fixed schedule)	77.7

Comparable stimuli in different people produce the same intensity of pain. (False)	76.0

The most likely explanation for why a patient with pain would request increased doses of pain medication is: (The patient is experiencing increased pain).	72.7

**Table 7:** Top 5 questions answered incorrectly by nursing students.

Question (correct answer)	% incorrect
A child with continuous or persistent pain has been receiving daily opioid analgesics for 2 months. The doses increased during this time period. Yesterday the patient was receiving morphine 20 mg/h intravenously. Today he has been receiving 25 mg/h intravenously for 3 hours. The likelihood of the patient developing clinically significant respiratory depression is: (less than 1%)	98.3

Narcotic/opioid addiction is defined as psychological dependence accompanied by overwhelming concern with obtaining and using narcotics for psychic effect, not for medical reasons. It may occur with or without the physiological changes of tolerance to analgesia and physical dependence (withdrawal). Using this definition, how likely is it that opioid addiction will occur as a result if treating pain with opioid analgesics? (<1%)	97.7

Your assessment, above, is made 2 hours after he received morphine 2 mg IV. Half-hourly pain ratings after the injection ranged from 6 to 8, and he had no clinically significant respiratory depression, sedation, or other side effects. He has identified 2 as an acceptable level of pain relief. His physician's order for analgesia is “morphine IV 1–3 mg q 1 h PRN pain relief”. Check the action you will take at this time: (Administer morphine 3 mg IV now).	96.3

The recommended route of administration of opioid analgesics to children with continuous or persistent pain is: (oral)	91.3

What do you think is the percentage of patients who over-report the amount of pain they have? Circle the correct answer: (0 to 10 %)	91.0

**Table 8:** Nursing students knowledge about which of the following drugs are or are not opioid or opiate.

	Correct responses	
	*n*	%
Morphine	241	80.3
Fentanyl	103	34.3
Ibuprofen	134	44.7
Ketorolac	166	55.3
Metamizol	174	58.0
Nalbuphine	137	45.7
Naproxen	169	56.3
Nimesulide	120	40.0
Paracetamol	171	57.0
Indomethacin	108	36.0
Tramadol	93	31.0
Aspirin	172	57.3
